# Psoriasis Flare-Up in a Patient Treated With Docetaxel for Metastatic Prostate Cancer

**DOI:** 10.7759/cureus.34726

**Published:** 2023-02-07

**Authors:** Matthew Kurian, Kanchi Patell, Kanithra Sekaran

**Affiliations:** 1 Hematology and Medical Oncology, University Hospitals Cleveland Medical Center, Cleveland, USA; 2 Internal Medicine, MetroHealth Medical Center, Cleveland, USA

**Keywords:** management of psoriasis, skin eruption, drug-induced psoriasis, chemotherapy-related toxicity, castration-resistant metastatic prostate cancer

## Abstract

Dermatologic toxicities, such as urticaria and mucositis, with docetaxel, have been commonly reported; however, fixed-plaque erythrodysesthesia is a rare adverse phenomenon with a reported incidence of less than 5% of patients. Docetaxel-induced psoriasis is extremely rare, and to date, very few cases have been reported in the literature. We present a literature review of psoriasis cases secondary to docetaxel and report our own case of severe docetaxel-induced psoriasis in the setting of treatment of metastatic prostate cancer. Our patient received topical steroids and narrow-band ultraviolet B (NBUVB) light therapy with resolution of their psoriasis and was able to complete their chemotherapy without discontinuation or interruption of their docetaxel.

## Introduction

Docetaxel was first extracted in 1986 from the needles of the European Yew tree (Taxus baccata) [1]. It belongs to the taxane class of chemotherapeutic agents and exerts its cytotoxic effect by disrupting the microtubular network in cells. Docetaxel binds to the b-subunit of tubulin, causing tubulin polymerization and inhibiting microtubule depolymerization. As a result, the cell cycle is arrested at the G2/M phase, and mitosis is inhibited, leading to cell death [2,3]. Docetaxel has been Food and Drug Administration (FDA) approved to treat a wide variety of solid tumors, such as breast cancer, non-small cell lung cancer, hormone-refractory prostate cancer, gastric adenocarcinoma, and squamous cell carcinoma of the head and neck [4]. It is administered over one hour at a dose of 75 mg/m^2^ every three weeks. The most reported toxicities of docetaxel include fatigue, myalgia, arthralgia, nausea, neutropenia, and peripheral neuropathy [5].

## Case presentation

**Figure 1:**
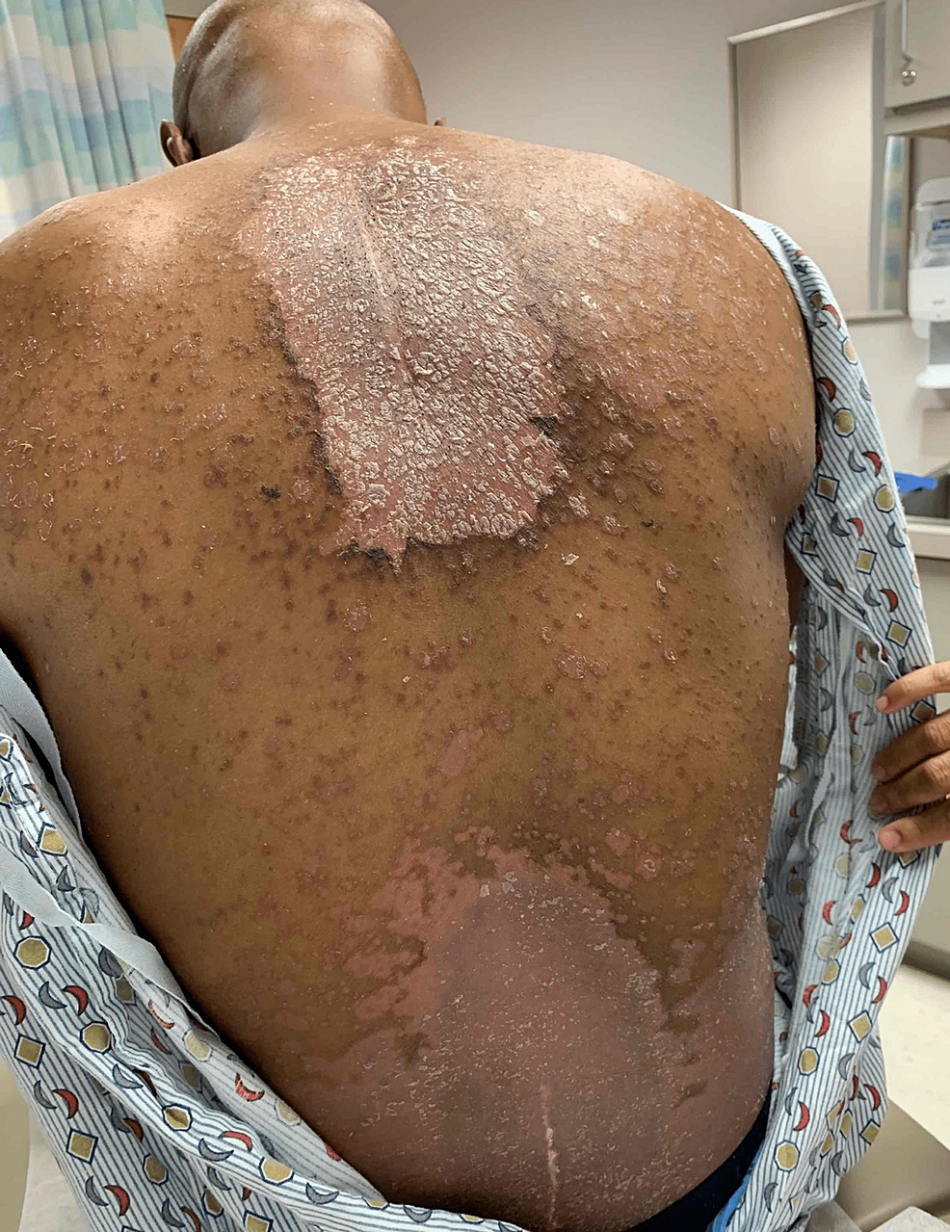
Rash on Day 7 of Docetaxel (Back)

**Figure 2:**
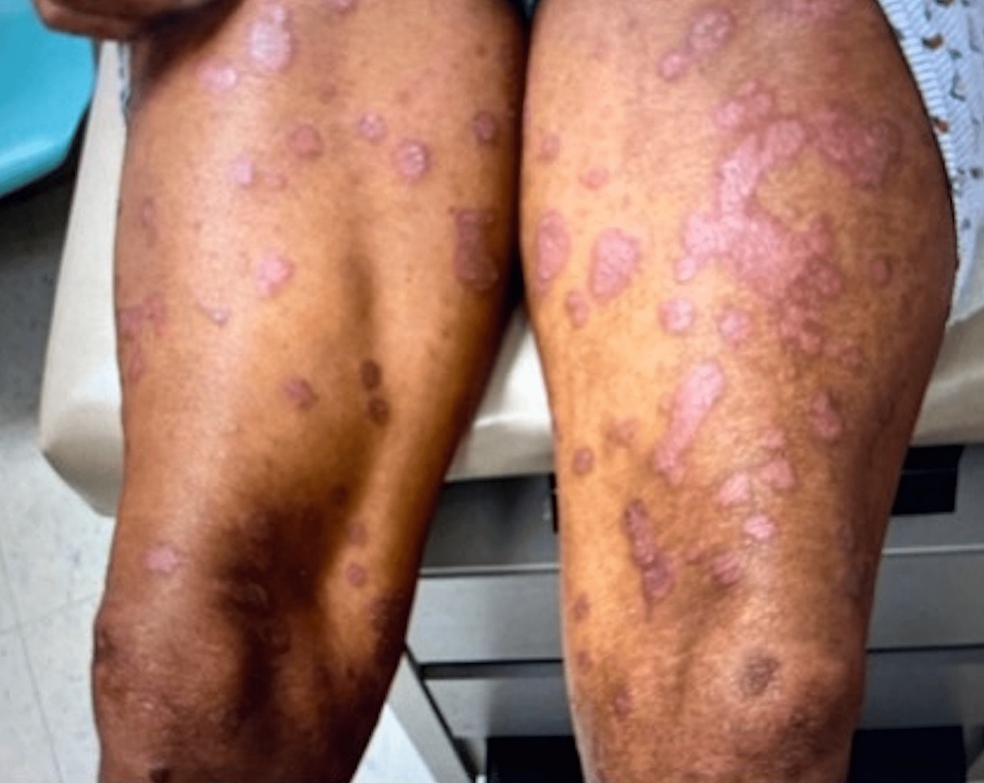
Rash on Day 7 of Docetaxel (Anterior Thighs)

**Figure 3:**
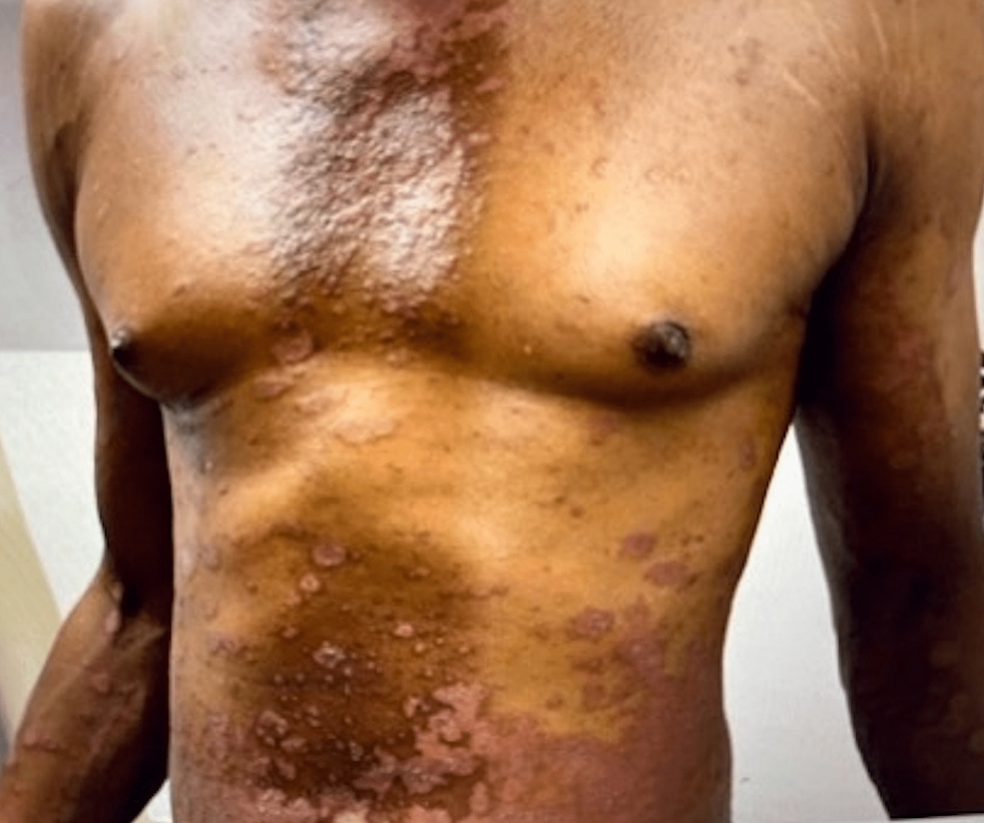
Rash on Day 7 of Docetaxel (Chest)

**Figure 4:**
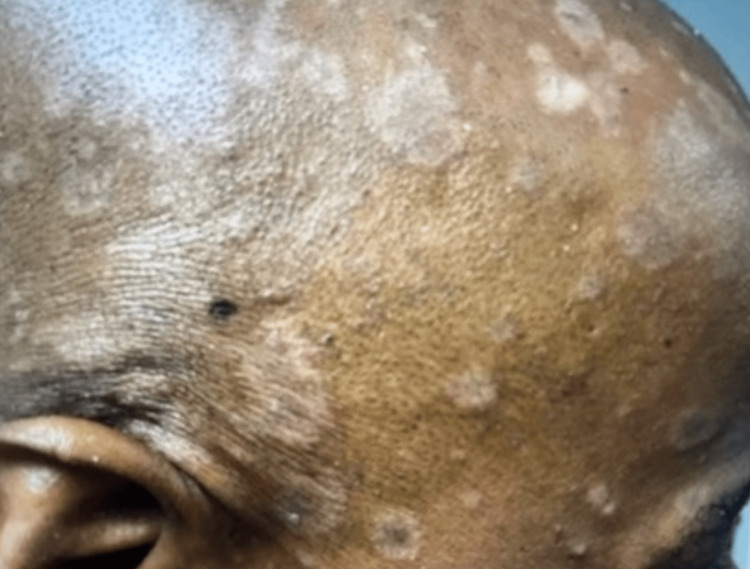
Rash on Day 7 of Docetaxel (Right Head)

**Figure 5:**
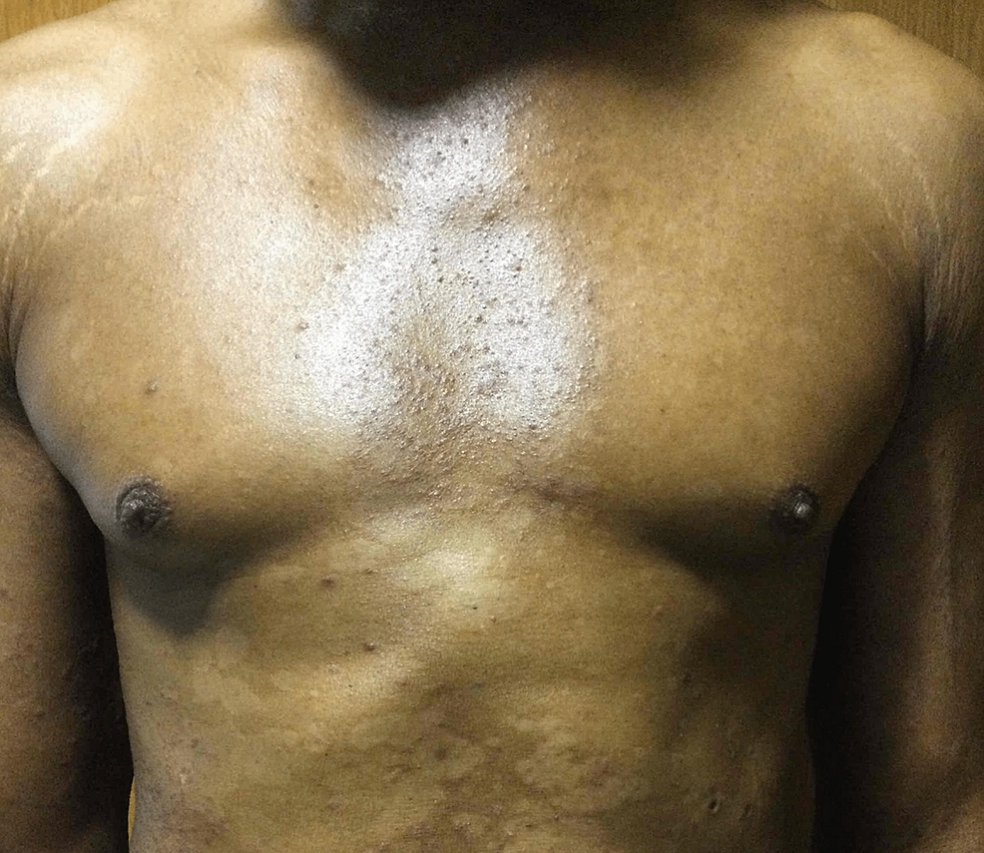
Rash on Completion of Cycle 6 of Docetaxel (Chest)

**Figure 6:**
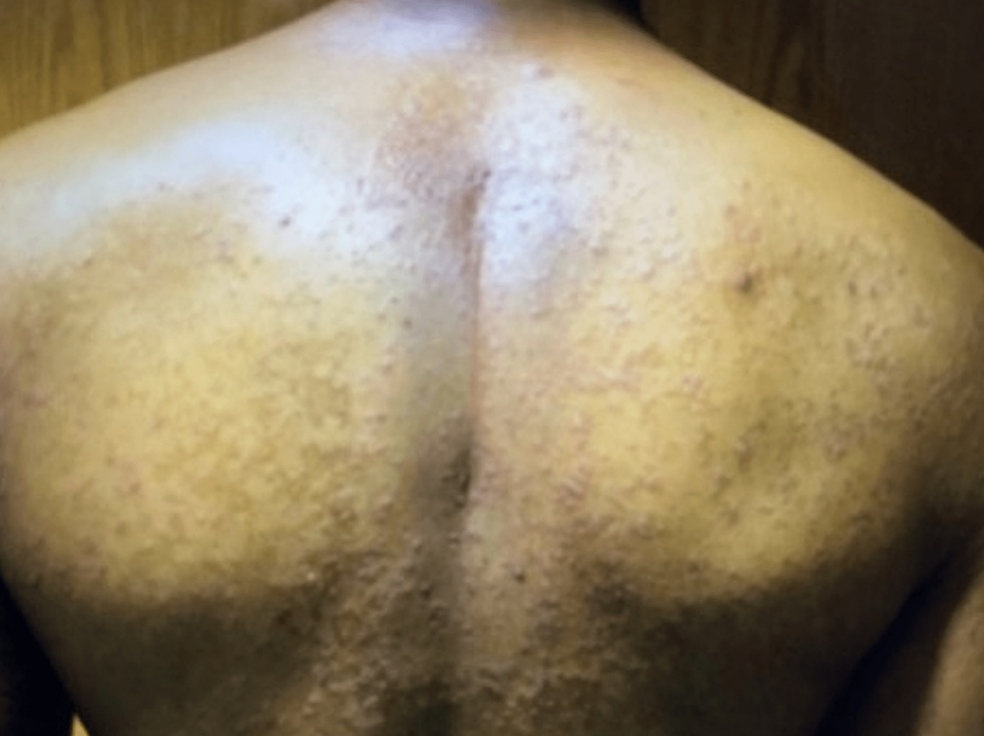
Rash on Completion of Cycle 6 of Docetaxel (Back)

The patient was a 47-year-old African American male with a remote history of psoriatic arthritis diagnosed twenty years ago who initially presented with worsening bilateral extremity weakness, saddle anesthesia, and fecal and urinary incontinence consistent with cord compression on MRI imaging. His prostate-specific antigen (PSA) was measured to be > 1500, and he subsequently underwent an emergent decompression of L5-S1 vertebrae. He then was subsequently started on oral bicalutamide daily for seven days and then received an injection of leuprorelin shortly after. Given his overall high burden of metastatic prostate cancer, he was transitioned to docetaxel 75 mg/m^2^ every three weeks with the goal of six cycles of chemotherapy. Seven days after the completion of his first cycle of chemotherapy, the patient then developed a severe diffuse plaque-like rash covering his body (Figures 1-4). He was urgently referred to dermatology, who recommended a biopsy of one of his new skin lesions, which was consistent with psoriasis. The patient had a remote history of mild psoriatic arthritis in his early 20s that responded previously to low-dose topical corticosteroids and never had a flare until this presentation. He was then initiated on triamcinolone ointment 0.1% twice daily to the affected areas with mild relief. Dermatology subsequently added calcipotriene cream twice daily to affected areas and NBUVB light therapy twice per week. Given that the patient was young and seeking finite treatment of his cancer with six cycles of docetaxel as opposed to life-long treatment, it was determined by oncology that the chemotherapy should be continued at a full dose to maximize treatment benefit. The patient’s psoriasis improved dramatically with a resolution of 75% of lesions, and he finished chemotherapy with successful control of his metastatic prostate cancer and no further new psoriasis lesions (Figures 5, 6). In terms of his psoriatic arthritis, the patient did not have any arthralgias, and his psoriasis was well controlled on steroids and calcipotriene.

## Discussion

There have been only three case reports published in the literature on patients with docetaxel-induced psoriasis. 

Watabe et al. described an 80-year-old woman with no history of psoriasis who received docetaxel for the disease progression of her lung adenocarcinoma after conventional chemotherapy. After four cycles of therapy, she started developing scaly erythematous lesions over her trunk and extremities. Histological examination of a skin biopsy specimen revealed hyperkeratosis, elongation of the rete ridges, and neutrophilic infiltration below the stratum corneum. All laboratory work-up was negative, and based on the physical and histological examination, she was diagnosed with psoriasis. After discontinuation of docetaxel and treatment with topical corticosteroids and calcipotriol, a gradual resolution was achieved in six to eight weeks [6].

Yang et al. described a 78-year-old man who received his first dose of 90 mg/m2 of docetaxel for the treatment of his prostate cancer. He had a 50-year history of well-controlled psoriasis confined to hands and feet with the use of topical agents. Three days after receiving docetaxel, the patient was noted to have an exacerbation of their psoriasis. The psoriasis area severity index was 10.4, and 20% of his body surface area was involved. The patient was managed as psoriasis exacerbation secondary to docetaxel, which was discontinued. He was treated with acitretin at a dose of 20 mg daily and topical calcipotriol/betamethasone dipropionate. An improvement in psoriasis lesions began after 10 weeks of treatment [7].

Allende et al. reported a case about a 65-year-old male with a family history of psoriasis who began receiving docetaxel (treatment due to the progression of squamous cell carcinoma of the lung). He developed scattered lesions on the trunk and extremities eight days after receiving docetaxel. The lesions were punctate, erythematous, and scaly. A skin biopsy showed the epidermis with psoriasiform hyperplasia, acanthosis, parakeratosis, elongation of the interpapillary ridges, isolated lymphocytes, and a polynuclear infiltrate, and more superficial epidermal strata forming Kogoj spongiform pustules. In the dermis, a perivascular inflammatory infiltrate associated with dilated capillaries was observed. Both findings in the epidermis and dermis were consistent with psoriasis. The psoriasis was well controlled with topical corticosteroids and calcipotriol. Unfortunately, the patient had another exacerbation of his psoriasis with the subsequent cycle of docetaxel [8]. 

Psoriasis is an immune-mediated dermatosis, and its etiology in relation to docetaxel administration is still unclear. However, it is thought to be due to an alteration in the immune environment secondary to cytotoxic chemotherapy. One theory suggests docetaxel leads to a reduction in regulatory T cells (Treg) in the peripheral blood. Treg cells are responsible for altering the expressions of cytokines such as IFN-a, which is known to play a role in psoriasis. In addition, imbalance and impaired Treg cells have been found in psoriasis patients [9,10]. The improvement in psoriasis after discontinuation of docetaxel chemotherapy supports the causal association between docetaxel and psoriasis aggravation.

Typical management of psoriasis requires that patients be on long-term therapy as it is a chronic disease. Treatment choices are typically based on a multitude of factors, including disease severity, comorbidities, and access to health care [11]. Disease severity is divided into categories of mild to moderate and moderate to severe. Various severity scores are used clinically to assess disease severity, including the Psoriasis Area and Severity Index (PASI), Dermatology Life Quality Index (DLQI), Body Surface Area (BSA), and Physician Global Assessment Index (PGA) [12]. Mild to moderate psoriasis typically requires a combination of topical glucocorticoids, vitamin D analogs, and phototherapy, while moderate to severe psoriasis requires systemic treatment, which is traditionally immunomodulators such as methotrexate, cyclosporin A, and retinoids. More recently, systemic targeted therapies have entered as treatment options, including Apremilast and Tofacitinib, in addition to biologics such as TNF-α inhibitors, IL-17 inhibitors, and IL-23 inhibitors [13].

Due to the above-reported findings of docetaxel-induced psoriasis, the question of appropriate treatment strategy arises. The disease found in these patients is thought to be a mix of both new psoriasis and the re-emergence of underlying psoriasis. The first step would be a cessation of the offending chemotherapeutic agent, which may not solely resolve existing psoriasis skin lesions. Management of drug-induced psoriasis usually includes the typical treatment choices, including topical and systemic treatments [14]. Due to the possible immune environment modification of Docetaxel-induced psoriasis and underlying malignant conditions, biological agents are sometimes avoided for psoriasis treatment. In many reported cases, topical treatment with corticoids and vitamin D analogs was sufficient to obtain good results, but in more severe cases, topical treatments alone were insufficient, and patients required oral prednisolone, acitretin, or phototherapy [15]. In patients who require continued treatment with Docetaxel, such as the patient in our case, immunosuppressants may be reinstated cautiously after adequate control of psoriasis symptoms is achieved. 

## Conclusions

Psoriasis secondary to docetaxel is rarely reported in the literature. Our case suggests that careful monitoring is needed to detect psoriasis following docetaxel administration in patients with and without a history of dermatologic disease. Docetaxel can cause a variety of dermatological side effects, including psoriasis. Flares in those with and without pre-existing psoriasis can occur one to two cycles after initial docetaxel introduction. Patients should also regularly be made aware of psoriasis as a side effect to surveil while they are receiving chemotherapy. The preferred method of treatment is the discontinuation of docetaxel. Dermatologists should also be consulted as early as possible after the identification of psoriasis symptoms. However, in our case, the patient wanted a finite amount of chemotherapy to be given as opposed to the lifelong treatment for his stage 4 prostate cancer, which warranted our approach to continuing chemotherapy and treating his psoriasis aggressively. Corticosteroids are the first line for psoriasis, but a patient may require additional therapies such as Vitamin D analogs like calcipotriene and UV light therapy in refractory cases. In summary, docetaxel chemotherapy can lead to psoriasis, and prompt dermatological consultation and co-management are key to the successful management of this rare side effect.
